# Natural course of adult-onset vitelliform lesions in eyes with and without comorbid subretinal drusenoid deposits

**DOI:** 10.1007/s10792-020-01319-2

**Published:** 2020-03-04

**Authors:** Craig Wilde, Mary Awad, Konstantinos Giannouladis, Arun Lakshmanan, Aaron Ming-Hon Yeung, Harminder Dua, Winfried M. K. Amoaku

**Affiliations:** 1 Ophthalmology and Vision Sciences, Division of Clinical Neurosciences, B Floor, EENT Centre, Queen’s Medical Centre, University of Nottingham, Nottingham, UK; 2 Nottingham University Hospitals NHS Trust, Nottingham, UK

**Keywords:** Adult-onset vitelliform lesions, Subretinal drusenoid deposits, Reticular drusen, Pseudodrusen, Prognosis, Adult-onset foveomacular vitelliform dystrophy

## Abstract

**Purpose:**

Adult vitelliform lesions (AVL) are associated with age related macular degeneration (AMD) and subretinal drusenoid deposits (SRDD). We evaluated the natural course of AVL, assessing the influence of SRDD on disease progression, visual function and incidence of macular atrophy (MA) and choroidal neovascular membranes (CNVM).

**Methods:**

A retrospective cohort study was conducted between January 2011 and March 2016. Demographic, clinical and imaging data from 26 consecutive AVL patients were analysed following case note review. Optical coherence tomography images were graded for SRDD and patients divided into those with/without SRDD. Outcomes included presenting/changes in best corrected visual acuity (BCVA) and incidence of MA/CNVM.

**Results:**

Mean age was 78.6 ± 7.6 years. Mean follow-up was 51.5 ± 25.6 months. Twelve patients (46.2%) had SRDD at presentation with 3 more (11.5%) developing them. Subjects with SRDD were older (mean 81.7 ± 6.1 years vs 74.3 ± 7.6 years, *p* = 0.010). Mean presenting BCVA was worse in SRDD eyes (0.39 ± 0.31 logMAR vs 0.19 ± 0.18 logMAR, *p* = 0.017). Eight of 15 patients with SRDD (53.3%) developed incident MA or CNVM; higher than those with no SRDD (1/11, 9.1%; *p* = 0.036). Two patients (7.7%) developed full thickness macular holes.

**Conclusions:**

Patients with AVL and SRDD likely represent an advanced pathological stage or phenotype with worse visual outcome and higher risk of MA/CNVM. Possible overlap with AMD exists. Follow-up, counselling and provisions for early detection/treatment of complications should be made. Better classification including improved understanding of phenotypic and genetic variations with reference to comorbid diseases including AMD is required. Presence of SRDD in AVL offers a dichotomous classification, indicating risk of future MA/CNVM formation.

## Introduction

Adult-onset foveomacular vitelliform dystrophy (AFVD) is a relatively uncommon macular disease, initially described by Gass in 1974 [1]. It has several phenotypic similarities with Best vitelliform macular dystrophy, including typical bilateral subretinal deposition of yellowish material at the macula. It has traditionally been included among a heterogeneous group of pattern dystrophies thought to be of inherited origin, but there is ongoing debate regarding the role of genetics in its aetiology. Possible overlap between the condition and age-related maculopathy has led some investigators to believe that for the majority of cases, AFVD is degenerative, but perhaps with a genetic predisposition. Multiple studies (including the original description by Gass) report the presence of comorbid drusen of various morphologies and also more recently subretinal drusenoid deposits (SRDD) [1–4]. The condition is heterogeneous, with highly variable patterns of inheritance, clinical appearance and course, with variable age of onset. It often remains asymptomatic until the fifth decade. Phenotypes can vary depending on when the disease is studied and patient age. Long-term visual prognosis varies among individuals. For most, the clinical course is benign, but for others, particularly older individuals, central vision can be substantially reduced secondary to macular atrophy (MA) and/or development of choroidal neovascular membrane (CNVM) [5]. Factors that predict a phenotype that poses greater risk of visual loss or phenotypic features that cause more rapid progression to MA or incident CNVM are difficult to study given the variability. However, certain features have been associated with visual reduction, including retinal thickness and lesion stage, as determined by optical coherence tomography (OCT) [6]. Some have used spectral-domain (SD) OCT to quantitatively link visual loss with disruption to the ellipsoid zone [7].

We recently reported SRDD are a frequent finding in eyes with newly presenting AFVD, not being restricted to AMD, but common among diseases where pathophysiological mechanisms involve damage to Bruch’s membrane and RPE, whether genetic or degenerative [2]. With this in mind, we analysed the natural course of newly presenting patients with the clinical diagnosis of AFVD within our department, reviewed structural and functional changes over time and assessed the influence SRDD may have on disease progression, incidence of CNVM/MA and visual loss. Because of a lack of genetic testing and no universal availability of family history, we use the broadly descriptive term adult vitelliform lesions (AVL) throughout this manuscript to describe our patient cohort.

## Materials and methods

All consecutive patients evaluated in our department with newly presenting AVL (diagnosed by retinal specialists AL, AY, CW and WA) between January 2011 and March 2016 were retrospectively reviewed throughout their longitudinal clinical follow-up. All available case notes reviewed and multimodal images were graded. All patients underwent complete ophthalmological examination in clinic including measurement of best corrected visual acuity (BCVA) as evaluated with a LogMAR chart (or in some cases a Snellen chart), mydriatic fundus biomicroscopy, SDOCT, colour fundus photographs (FP), fundus autofluorescence (FAF) and fundus fluorescein angiography (FFA) as indicated. For study purposes, Snellen BCVA was converted to logMAR for analysis.

**Fig. 1:**
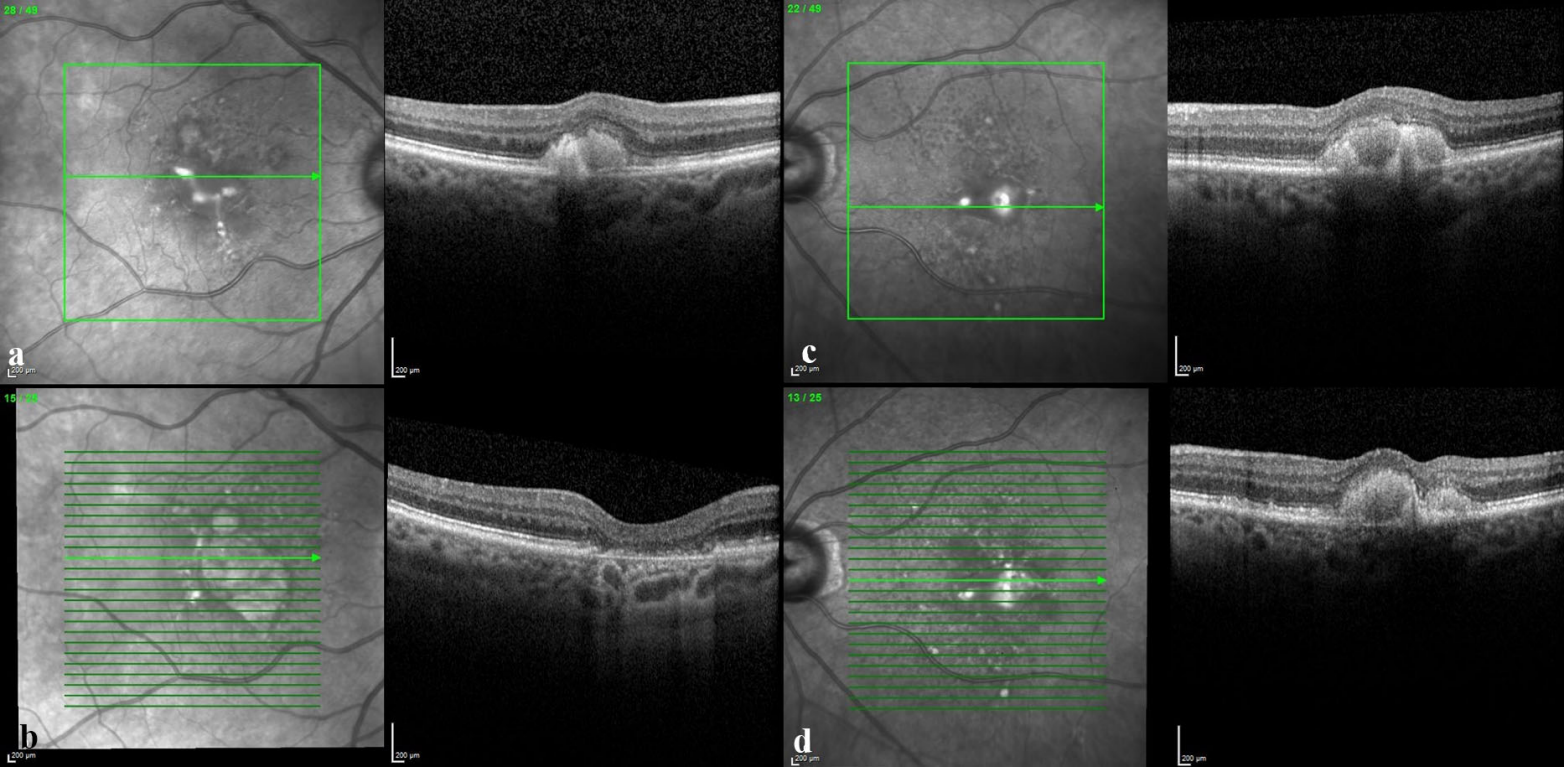
Montage of SDOCT images of a 74-year-old female presenting in 2012 with bilateral AVL. LogMAR BCVA was 0.3 in the right eye (**a**) and 0.2 in the left (**c**). Five years later, the right AVL had regressed to leave macular atrophy with corresponding decrease in BCVA to LogMAR 1.0 (**b**). The left eye LogMAR BCVA decreased to 0.7 (**d**). SRDD are visible within the en-face images in the immediate perilesional areas, particularly within the superotemporal macula. Over the 5-year follow-up period, SRDD increase in number, spreading towards the vascular arcades, particularly superiorly. The ISel can be seen to fragment and become irregular in the left eye between presentation (**c**) and follow-up (**d**)

Criteria for diagnosis of AVL were the presence of round or oval, more or less homogenous yellowish sub-/perifoveal lesions at fundus examination with the corresponding presence of a hyper-reflective dome-shaped subretinal material on SDOCT (Fig. 1a–c) and hyper-autofluorescence. The unit of study for calculations and measurements were adult patients with AVL in at least one eye. Exclusion criteria included eyes with poor-quality images preventing adequate grading and eyes with comorbid ocular pathologies (at presentation) known to be associated with phenocopies of vitelliform lesions, including vitreomacular traction (VMT) and epiretinal membranes (ERM). If such lesions were a unilateral finding, with AVL in both eyes, or, where such conditions developed during temporal follow-up, the patient was included in the study. To allow real-world representation of AVL patients, subjects with AVL in one eye and signs of CNVM or geographic atrophy (GA) at presentation in their contralateral eye were included in the study. Where such lesions prevented grading of vitelliform lesions, eyes were removed from analysis. No genetic testing was performed on the population, and no electrooculograms were undertaken. Eyes with drusen were not excluded (given their high prevalence within this age group) [8], unless they were the dominant clinical feature with suspicion of a central drusenoid pigment epithelial detachment. No family history data were recorded for the majority of patients.

All eyes with AVL (at presentation) were graded for the presence or absence of SRDD using SDOCT images acquired using either the 3D OCT-1000 instrument (Topcon, Tokyo, Japan) or the Spectralis SDOCT (Heidelberg Engineering, Heidelberg, Germany), with the department transitioning between the two during the study period. SRDD were graded if there were five or more discrete hyper-reflective collections in the subretinal space, above the retinal pigment epithelium (RPE), being sufficient to alter the contour of the inner segment ellipsoid (ISel) in a saw tooth pattern. Field of image acquisition for the 3D OCT 1000 was a 6 mm by 6 mm area centred on the fovea. Scanning protocol consisted of 128 frames each made up of 512 axial scans for each eye. Spectralis images were acquired using a 25-horizontal-line protocol (using a 6 mm by 6 mm area). All frames were reviewed for the presence of AVL and SRDD by at least two authors (AL, CW or MA), with immediate open discussion. If disagreement existed, differences were adjudicated by all three graders.

**Fig. 2:**
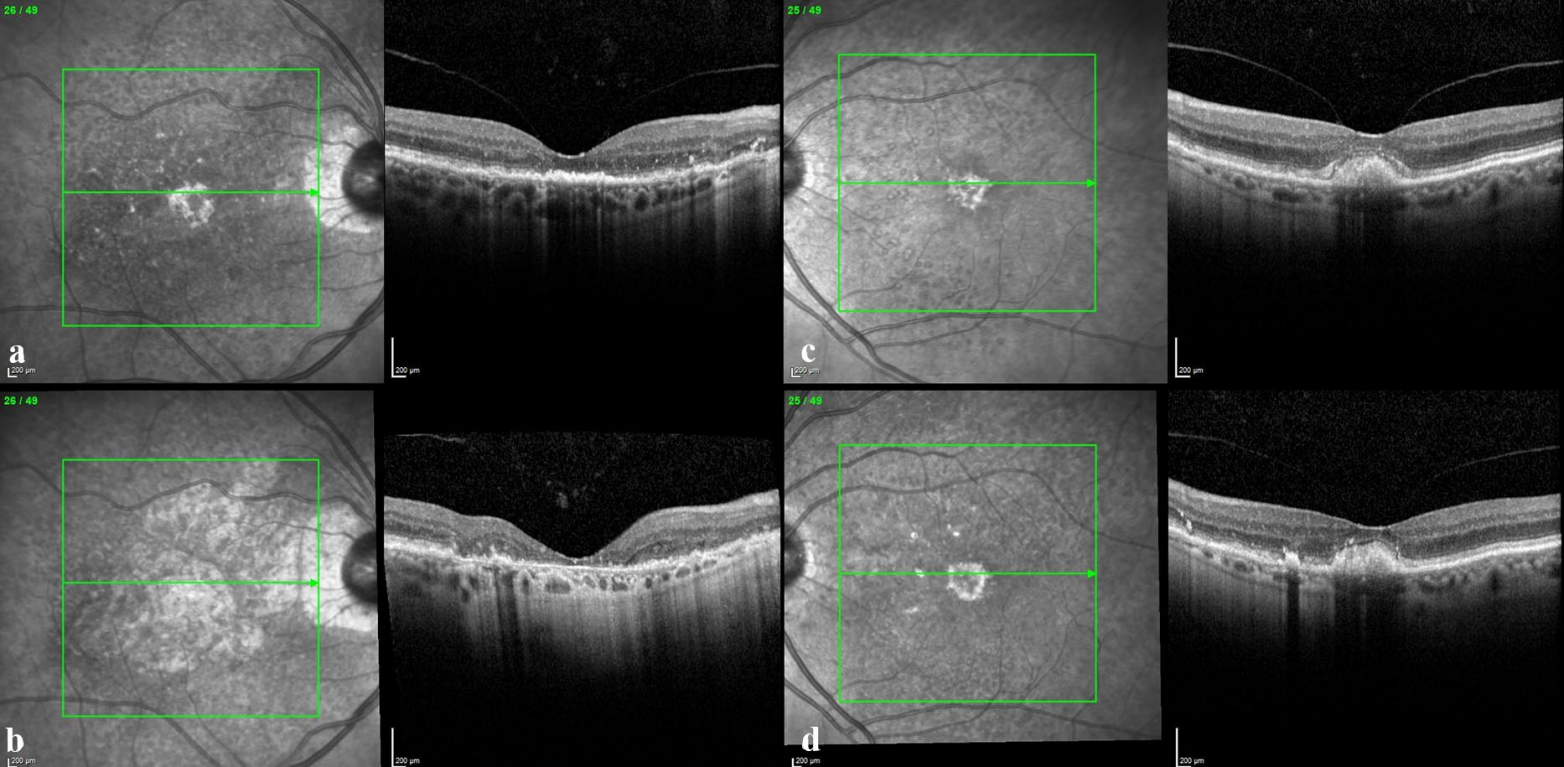
A 75-year-old female presents in 2011 with bilateral AVL and comorbid SRDD. Advice is given and the patient discharged. In 2015, they re-present with reduced vision (LogMAR 1.0 and 0.5 in the right and left eyes respectively). The right AVL (**a**) has regressed, and there is widespread outer retinal atrophy with loss of the ISel, thinning and irregularity of the RPE, with pigment migration towards the inner retina (**a**). There is bilateral vitreomacular adhesion. The left eye (**c**) shows fragmentation and thinning of the ISel as it travels over the apex of the AVL. Two years later, BCVA has decreased further (CF and LogMAR 0.68 in the right and left eyes, respectively) with widespread right eye macular atrophy (**b**). In the left eye, the number and area of SRDD had increased and there has been further thinning and fragmentation of the ISel over the AVL. This case highlights asymmetry between the two eyes. It was noted that at presentation, although bilateral, SRDD were more numerous in the right eye. The spatiotemporal relationship between SRDD area and AVL morphology and evolution remains to be studied

Macular atrophy (MA) was diagnosed using all available imaging from clinics. For the grading of fundus photographs, MA was defined as a sharply demarcated area of RPE loss that was at least 175 µm in diameter being roughly round or oval in shape, with at least 2 of the following features: scalloped edges, visible choroidal vessels that are more prominent than in the surrounding areas and well-defined margins in keeping with the clarity of the photograph. Using SDOCT, MA was defined as a sharply demarcated area of degeneration of the RPE and outer retina with the corresponding increased reflectivity within the underlying choroid (Figs. 1b, 2b). Sharply demarcated outer retinal atrophy with the defined loss of the ISel with underlying thinning and fragmentation of the RPE was also included among the definition. These changes could be located anywhere within the 6 mm by 6 mm macular grid, including eccentric lesions.

Patients were divided into two groups: AVL with or without SRDD at presentation. All subsequent visits and re-presentations were analysed in a longitudinal fashion. All images were analysed for development of either MA or CNVM. BCVA was recorded for each visit.

Data acquired from case note review included age, gender and comorbid ocular diseases. Patient follow-up visits varied according to clinical need, with some patients being discharged with an open appointment if problems developed or clinical need arose. Results of clinical assessments and outcomes were reviewed from each visit.

Outcomes measured included mean BCVA and change in BCVA in comparison with baseline visit for eyes with or without SRDD and incidence of MA and CNVM for each. Secondary outcomes were rates of re-presentation to hospital eye services.

Statistical analysis was performed using statistical package for the social sciences (SPSS) for Mac version 24 (SPSS Inc., Chicago, Illinois, USA). Results were presented as proportions (%) for categorical variables and mean ± standard deviation (SD) for continuous variables. The Student t test was used to compare continuous data. Unpaired t test was used to evaluate and compare baseline differences between AVL with and without SRDD for continuous variables including baseline logMAR BCVA and period of follow-up. Mean changes in BCVA were compared with the unpaired Student t test. For binary outcomes, including incidence of MA/CNVM, Fisher’s exact test was used for intergroup comparisons of proportions. P values of 0.05 or less were considered statistically significant.

Ethics committee approval was not required for this study, and it adhered to ethical principles outlined in the declaration of Helsinki.

## Results

### General

A total of 26 Caucasian patients with newly diagnosed AVL were included in the study. Mean age at presentation was 78.6 ± 7.6 years (range 63–96 years). The cohort was composed of 13 female subjects (50%) and 13 males (50%). Nine patients (34.6%) received no follow-up, being discharged at their first visit and did not re-present to the hospital eye service (HES). A total of two patients received initial hospital follow-up (one with CNVM and one with suspected glaucoma), with a further 15 patients re-presenting to the HES (57.7%). Mean follow-up duration was 51.5 ± 25.6 months (range 9.8–84.1). Mean BCVA at presentation was 0.32 ± 0.29 logMAR (range − 0.04–1.2). Where follow-up was available, mean change in BCVA was − 0.19 ± 0.22 logMAR (range − 0.7 to 0.3). Five (5) individuals presented with a BCVA of worse than 0.3 logMAR in their better seeing eye (19.2%). Twelve (12) patients had SRDD at presentation (46.2%) with a further 3 developing SRDD during longitudinal follow-up (15/26 in total, 57.7%).

### Incident geographic atrophy and choroidal neovascularization

Four (4) of the 26 patients (15.4%) presented with evidence of either CNVM or MA in either eye. All of these patients had SRDD. Two patients had MA (which was eccentric and not foveal involving), one had CNVM, and the fourth had a disciform scar. No patients without SRDD presented with MA or CNVM. Throughout follow-up, seven patients developed MA (26.9%), with bilateral incident MA in 3 of these individuals. One of these patients had a pre-existing disciform scar contralaterally. Two patients developed incident CNVM (7.7%), being bilateral in one of these individuals. All eyes (with CNVM) received treatment with an intravitreal anti-vascular endothelial growth factor, and at the final visit BCVA was 0.33 ± 0.23 logMAR (range 0.2–0.6). Overall patients with AVL have a significant risk of either presenting with or developing incident CNVM during their follow-up (4/26, 15.4%) with an even higher risk of MA development (9/26, 34.6%).

Out of the 15 patients with AVL that presented with SRDD or developed them during longitudinal follow-up, 8 subjects (53.3%) developed incident MA or CNVM. Only one patient out of eleven who had AVL and no SRDD developed incident MA/CNVM (9.1%). This difference was deemed statistically significant (*p* = 0.036, Fisher’s exact test). Eleven of the 15 eyes with AVL with SRDD at presentation (or those that would develop SRDD during follow-up) either presented or would develop MA or CNVM in either eye (73%) compared to one of 11 patients (9.1%) without SRDD (*p* = 0.0017). Two of the 15 subjects with AVL and SRDD developed incident CNVM (13.3%), one with bilateral disease, compared to nil subjects with isolated AVL (*p* = 0.49).

Mean age of subjects with AVL and SRDD was 81.7 ± 6.1 years (range 71–96 years) while those with AVL and no SRDD were generally younger with mean age of 74.3 ± 7.6 years (range 63–85 years), a difference which was statistically significant (*p* = 0.010). Of the 15 patients with AVL and SRDD, 11 patients re-presented to the HES or were followed up, (73.33%) while 6 of the 11 patients (54.5%) with AVL and no SRDD re-presented (*p* = 0.42).

### Visual acuity

Patients with AVL and SRDD had a mean BCVA of 0.39 ± 0.31 logMAR, which was worse than that for those with no SRDD, who had a mean BCVA of 0.19 ± 0.18 logMAR. The difference was statistically significant, *p* = 0.017.

Where BCVA data were available, mean change in BCVA in the AVL and SRDD group was − 0.23 ± 0.23 logMAR (22 eyes). For the AVL and no SRDD group, the change in BCVA was − 0.12 ± 0.17 logMAR (10 eyes). The difference was not statistically significant (*p* = 0.20). Where follow-up or re-presentation occurred, the duration for the two groups was 60.2 ± 19.5 months vs 39.2 ± 31.4 months (*p* = 0.12).

Of the three eyes that developed CNVM during follow-up and were treated with a course of intravitreal ranibizumab injections, mean change in BCVA was − 0.21 ± 0.023 logMAR (range − 0.24 to − 0.2). Mean duration of follow-up for these eyes was 39.3 ± 19.6 months (range 28–62). Of the nine eyes that developed MA, mean change in BCVA was − 0.29 ± 0.28 logMAR (range − 0.5 to 0.3). One eye had visual gain following cataract surgery. Mean duration of follow-up for these eyes was 66.6 ± 13.0 months (range 47–81).

During the follow-up period, two patients developed full thickness macular holes (7.7%).

## Discussion

Our findings confirm previous reports that the majority of cases of AVL are diagnosed in elderly individuals (mean age 78.6 years), with an equal gender distribution, the majority of whom present with good levels of vision. Upon initial diagnosis 80% of patients will achieve a Snellen BCVA in their better seeing eye of 6/12 or better, indicating initial early presentation with mild levels of visual dysfunction, often with asymmetry. Our findings suggest that for a third of patients following initial review, diagnosis and discharge, the condition remains largely benign, with no significant subjective change causing concern to warrant re-presentation to local HES. For the majority, however (two thirds), the condition is slowly progressive and new issues regarding symptoms, clinical findings or visual decline results in re-referral. Where follow-up or re-presentation takes place, for the majority, decline in BCVA is mild. In our study, with a long mean duration of follow-up of 4.3 years, mean loss of vision equates to approximately two ETDRS lines. Significant binocular visual loss is uncommon.

The development of CNVM in eyes with AVL is a recognised complication, with a varied incidence reported. Limited data exist on phenotypes with a predisposition for higher incidence. In one study of eyes with acquired vitelliform lesions, those with changes associated with comorbid age-related macular degeneration (AMD) had an increased risk of CNVM (12.4%) compared to those with isolated vitelliform lesions (2.1%) [9]. However, in a series of sporadic cases of AVL, Tiosano et al. report the conflicting finding that AVL eyes with or without drusen were similar in terms of lesion morphology, demographics and disease progression [10]. We report that overall patients with AVL have a significant risk of either presenting with or developing incident CNVM during their follow-up (15.4%) with an even higher risk of MA (34.6%). Furthermore, we report the finding, for the first time, that eyes with AVL and comorbid SRDD were statistically more likely to develop incident CNVM/MA when compared to those without SRDD. The risk of having or developing MA or CNVM is very high (over 70%), if SRDD are present or develop, offering a convenient and dichotomous approach to risk stratification, without the need for genotyping or other expensive testing. This is not surprising given SRDD have a prevalence that increases significantly with age [11] and they are a stronger predictor of AMD development than conventional drusen [12]. Eyes with AVL and SRDD were more likely to re-present and had a longer mean duration of follow-up, reflecting the higher incidence of MA and CNVM, resulting in greater symptoms and longer follow-up required for treatment. These findings are confounded by several issues, including the average older age of subjects with AVL and SRDD.

This study suggests the presence of SRDD may influence visual outcome among subjects with AVL. Mean presenting BCVA in eyes with AVL and SRDD was worse than subjects with no SRDD. They were also more likely to suffer greater visual decline, although the difference was not statistically significant.

Our finding that several patients presented with AVL, later developing incident SRDD, combined with older mean age and worse presenting BCVA, are highly suggestive that the phenotype reflects a more advanced stage of the same pathology, generally in older individuals, rather than a separate entity. Full genetic analysis would be required however, with testing for known genetic associations of both AMD and AVL. It is known that SRDD are a risk factor for the development of both MA and CNVM formation in AMD patients [12]. They are associated with outer retinal atrophy [13], visual dissatisfaction [11] and more recently poor visual outcome in eyes undergoing epiretinal membrane surgery [14]. Disruption of ISel is a known feature of SRDD. It is known that ISel integrity is correlated with preserved vision in eyes with AVL [10]. It is not surprising therefore that their presence in subjects with AVL increases the risk of MA, worse BCVA and higher incidence of CNVM. Our study reinforces shared characteristics between patients with AMD and AVL, including old mean age of presentation, association with drusen, SRDD and similar complications. Our findings confirm that for a large proportion of newly presenting AVL, there is a strong association with SRDD, the majority either having them or developing them. We cannot rule out that BCVA could be worse in the SRDD group as age is a confounder and patients may have more advanced cataracts, data for which are unavailable. However, upon representation, visually significant cataracts would or should have been addressed as clinically indicated by the treating physicians.

Several other interesting findings were noted. Eyes that developed incident CNVM maintained good BCVA when treated. The membranes may behave differently in their natural course compared to those associated with other aetiologies, or their early presentation and treatment or known tendency for neovascularisation to occur at the boundary of the vitelliform lesion may contribute to maintenance of good levels of vision [9]. The series is, however, too small to draw useful conclusions.

Full thickness macular holes (FTMH) have previously been reported as infrequent complications of juvenile [15] and adult-onset vitelliform macular disease [16]. Despite their rarity, they remain an important complication, requiring potential surgery, adding morbidity in subjects in whom they occur. Incidence has previously remained unreported. This study suggests it could be as high as 7.7%, being greater than previous reports may have indicated. However, the numbers involved are small and caution should be made with reference to this incidence. In contrast to idiopathic FTMH, where vitreomacular traction (VMT) is the primary aetiology, SDOCT studies suggest, in the setting of AVL, progressive retinal thinning results in development of a macular hole [16]. In our study, both patients had attached posterior hyaloid with visible vitreomacular adhesion. In one subject, the eye underwent a posterior vitreous detachment (PVD) and several months later a FTMH developed. In the second case, a PVD developed through a stage of VMT and the development of a FTMH. Vitreoretinal adhesion still plays a clear role in hole development. Holes may occur in weakened retina following vitelliruption of the lesion or during the normal process of PVD. It may be in some cases of AVL, eyes have an abnormally strong adhesion of the posterior hyaloid and weakened and thinned inner retina is more susceptible to breaks and FTMH formation. The true relationship between AVL, VMT and macular hole development has not been fully established and is likely via various pathways of VMT and outer retinal atrophy following PVD. The scenario of VMT causing AVL development as a phenocopy of true AVMD is a further complicating factor. In our clinical practice, we have seen eyes with SRDD and vitelliform lesions develop VMT, with PVD and formation of a lamellar hole. Subsequent outer retinal atrophy will result in the appearance of a full thickness macular hole. In these cases, PVD is generally responsible for inner retinal disruption and the formation of a lamellar hole.

The main limitations of this study are its retrospective nature and lack of follow-up for the minority of patients who were originally discharged without re-presentation. It is presumed these individuals did not have incident CNVM or MA. Unless these patients moved away or presented elsewhere, they would have likely been re-referred if significant pathology developed when alive. However, we cannot rule out the possibility of eccentric or asymptomatic disease that was foveal sparing going undetected, particularly if subjects did not attend optometry review in the community. Lack of genetic data is a limitation as it may have helped draw further distinctions between the heterogeneous groups of AVL, where clinical dividing lines between AVL, AMD and other conditions cannot be clearly drawn.

Although we present a continuous case series of newly presenting AVL, we cannot rule out misclassification of other patients not included here by treating clinicians, such as AMD. Some patients with AVL may have been classified as a drusenoid PED or AMD and missed from our series. Similarly, patients presenting with more advanced stages of AVL, without a typical AVL on OCT, particularly with bilateral atrophy, may have been misdiagnosed as GA and those at the vitelliruptive stage of the pathology may have been classified as other diseases including central serous retinopathy. For this reason, among others, we chose to include only lesions presenting with definite vitelliform lesions on SDOCT, where there is less ambiguity with reference to diagnosis.

Given the high incidence of CNVM/GA in eyes with SRDD, physicians could consider having a lower threshold for regular follow-up to recognise and manage any incident CNVM, particularly given the known difficulty in recognising this complication in eyes with AVL [9].

We find that the easily identifiable dichotomy between AVL that have and do not have SRDD appears to predict the risk of incident MA and CNVM formation without the need for expensive genotyping, utilising only SDOCT. We feel this finding has important clinical applications, making it easier to identify an at-risk subgroup.
